# Bariatric Surgery and Gender Differences in Depressive Symptoms—A 5‐Year Prospective Study of Preoperative Predictors

**DOI:** 10.1002/osp4.70120

**Published:** 2026-04-01

**Authors:** Liliana Buer, Ingela Lundin Kvalem, Tom Mala

**Affiliations:** ^1^ Department of Psychology University of Oslo Oslo Norway; ^2^ Department of Gastrointestinal and Pediatric Surgery Department of Endocrinology, Morbid Obesity and Preventive Medisin Oslo University Hospital University of Oslo Oslo Norway

**Keywords:** bariatric surgery, body image, depressive symptoms, gender, resilience

## Abstract

**Objective:**

Candidates for metabolic bariatric surgery (MBS) often exhibit a higher prevalence of depressive symptoms compared with the general population. Studies have shown improvements in depressive symptoms and a reduction in depression prevalence during the initial years following MBS. However, reports on the long‐term maintenance of these improvements are conflicting, and factors such as preoperative predictors and gender differences remain poorly understood.

**Methods:**

Data were collected from 210 subjects pre‐MBS and at 1‐ and 5‐years post‐MBS. Health care providers measured Body Mass Index (BMI). All other data were collected via self‐report (questionnaires). Pre‐MBS factors assumed associated with depressive symptoms at 5 years included BMI, body dissatisfaction, appearance orientation, resilience, and outcome expectancies.

**Results:**

The sample comprised 77.6% women. Pre‐MBS there were no significant gender differences in depressive symptomatology or the likelihood of being depressed. At both one and 5 years post‐MBS, a higher proportion of men were categorized as probably depressed. From baseline to 5 years post‐MBS, depressive symptoms declined among women, whereas there was no change among men. Regardless of gender, preoperative depressive symptoms and resilience predicted postoperative depression. Among women, preoperative body dissatisfaction and expectations regarding weight change and appearance were initially associated with postoperative levels of depression.

**Conclusions:**

Contrasting common findings, this study identified higher rates of post‐surgery depression in men than in women. Furthermore, the results indicate that post‐surgery depression may be bivariately associated with different factors depending on gender, although resilience predicted depressive symptoms irrespective of gender.

## 1 Introduction

Compared to the general population, individuals seeking metabolic bariatric surgery (MBS) typically exhibit a higher prevalence of depressive symptoms [1, 2, 3]. A growing body of research underscores that mental health improves within the first few years after MBS [4, 5, 6, 7, 8]. However, reports regarding the long‐term sustainability of post‐surgery improvements are more conflicting [5, 9]. To facilitate relevant post‐surgical support, additional research on long‐term mental health changes following MBS is needed. Particularly, longitudinal studies spanning 3 years or more remain limited in number [10]. Men and women may differ in psychological risk factors before MBS [11, 12]. However, few studies have explored gender differences related to depression longitudinally within the MBS context. Consequently, little is known about gender‐specific factors that might influence depressive symptoms post‐surgery.

Obesity has been linked to an elevated risk of depression, and conversely, depression has been identified as a risk factor for obesity [13]. However, this relationship does not appear strictly linear, and depressive symptoms after MBS have been empirically associated with diverse demographic, psychological, and social factors, as well as medical comorbidities [5].

Several review studies suggest a decline in the prevalence of clinical depression, as well as a reduction in the frequency and intensity of depressive symptoms, particularly within the first years following MBS [1, 5, 7, 14, 15, 16, 17]. Nevertheless, depressive symptoms tend to increase after the initial 2 years, although in most individuals, symptoms appear to remain improved compared to baseline [5]. Some studies have, however, noted a resurgence in depression symptomatology or an uptick in hospitalizations for depression two to 3 years post‐surgery [16, 18, 19, 20], indicating that depression rates were reverting to, or even surpassing, baseline levels six to 9 years following surgery [21, 22].

A higher prevalence of depressive symptoms and clinical depression among women compared with men has been found both in normal‐weight and over‐weight populations [11, 20, 23, 24, 25], as well as in patients seeking MBS [11, 12, 26, 27]. Higher rates of self‐reported depressive symptoms among women have also been reported post‐surgery [12, 28]. Additionally, a recent study [20] found that women exhibited higher rates of clinical depression before MBS, and a decrease in depression prevalence rates irrespective of gender during the first year after surgery, followed by an increase in the subsequent 2 years. A reduction in depressive symptoms following MBS, independent of gender, has been identified by other studies [29]. Additionally, Dixon, et al. [26]. noted a more pronounced decline in depressive symptoms among women 1 year after surgery.

While MBS typically leads to weight‐loss [30], a subset of patients experience weight regain [31, 32, 33]. Some studies have suggested that greater weight‐loss is associated with improved depression outcomes [16, 19, 34, 35], while others have reported mixed results, or no such link [5, 14, 36, 37, 38]. Additionally, reports have indicated that men typically present for surgery with a higher BMI than women [11, 12], and that women achieve greater weight‐loss [12], whereas others have found no significant gender differences [39, 40].

Various preoperative psychological factors may influence post‐surgical depressive symptoms. Research has found that pre‐MBS body dissatisfaction may predict depressive symptoms 6–12 months post‐surgery [41, 42]. While many studies have demonstrated a strong association between body image and weight/BMI [8, 10], changes in body weight do not always correspond with body satisfaction [37, 43]. Furthermore, studies suggest that the relationship between weight and depressive symptoms could be mediated by body dissatisfaction [44, 45, 46]. Women tend to exhibit higher levels of body dissatisfaction compared with men [12, 47, 48, 49, 50].

Appearance orientation, that is, an individual's investment in their physical appearance, has been associated with elevated levels of depressive symptoms [51]. In the general population, some studies have indicated that women score higher on appearance orientation compared to men [52, 53, 54], while others have found no such differences [55].

Negative expectations are often considered as a core feature of mental disorders [38] and are linked to depressive symptoms [43, 44]. Among candidates for MBS, many patients hold unrealistic expectations regarding weight‐loss [56, 57, 58]. Young candidates [56], particularly women, tend to have higher expectations for weight loss [56, 59] and for improved satisfaction with physical appearance [56]. Resilience, a cluster of factors known to protect against symptoms of mood disorders and maladjustment [60, 61], encompasses individual dispositions, along with sources of social support [62, 63]. In some studies, men exhibit higher levels of resilience [64], while others have found no gender differences [65]. The relationship between resilience and depressive symptoms has been confirmed cross‐sectionally prior to MBS and post‐surgically [66, 67, 68].

To enlighten these aspects, this study sought to address gender differences in depressive symptoms through a prospective longitudinal study and to investigate baseline factors that are assumed to be relevant for depressive symptoms after surgery. The first aim of this study was to examine gender differences in depressive symptoms before and 5 years after MBS and to investigate whether depressive symptoms changed differently depending on gender. The second aim was to identify, separately for women and men, pre‐surgery factors that may predict probable depression 5 years post‐surgery. To our knowledge, these relationships have not been prospectively investigated with outcome scores 5 years or longer after surgery, specifically focusing on gender differences. It was hypothesized that women would exhibit more depressive symptoms prior to, and after MBS, and that depressive symptoms would decline post‐surgically independent of gender.

## 2 Materials and Method

### 2.1 Participants and Procedures

The Oslo Bariatric Surgery Study is a prospective longitudinal cohort investigation. Participants were recruited during pre‐surgical consultations at the Oslo University Hospital over a 3‐year period (2011–2013). Following recruitment, postal questionnaires were distributed and collected by the Department of Psychology. Out of 506 eligible patients, 332 (66%) provided consent to participate in the study. Among these, 302 (91%) completed the baseline questionnaire. Subsequently, 16 participants withdrew from the study, of whom 12 did not proceed with the surgery. One year post‐surgery, 257 of the remaining 316 participants (81%) responded. At the 5‐year follow‐up (between 2016 and 2019), 222 of the 302 participants (74%) still engaged in the study and completed the questionnaire. This final study sample consisted of 210 participants who completed both the baseline and the 5‐year post‐surgery questionnaires. The participants had undergone either Roux‐en‐Y gastric bypass (*n* = 196) or sleeve gastrectomy (*n* = 14).

Attrition was analyzed using a multiple logistic regression analysis, incorporating all baseline study variables as predictors of non‐participation 5 years later. The analysis revealed no gender significant differences in drop‐out rates. However, participants who were not employed at baseline had a higher likelihood of dropping out of the study (OR = 2.19; 95% CI = 1.19–4.04, *p* = 0.012).

### 2.2 Measures

*Depressive symptoms* were assessed using the Hospital Anxiety and Depression Scale (HADS) [69], comprising seven items with response options ranging from 0 to 3. Sum scores were calculated at each time point (Cronbach's Alpha baseline = 0.78, 1‐year = 0.78, 5‐years = 0.80). High scores indicate more severe symptoms. Scores falling between 8 and 10 suggest mild symptoms, 11 to 14 indicate moderate symptoms, and 15 to 21 suggest severe symptoms [70]. According to the classifications of HADS, cut‐off scores of ≥ 10 indicate probable or possible depression, while scores below 10 suggest the absence of depression. In the text, these categories are referred to as “probable depression” and “no depression”.

*Body image* was assessed using two subscales from the Multidimensional Body Self Relations Questionnaire [71]: The Body Area Satisfaction Scale (BASS; 9 items, Cronbach's alpha = 0.73) and Appearance Orientation (AO; 14 items, only baseline, Cronbach's alpha = 0.85). Both scales utilized response categories ranging from 1 to 5. High mean scores on BASS indicate greater satisfaction with various body areas, weight, and overall appearance, while higher scores on AO reflect greater pre‐occupation with appearance.

*Expectations* were measured at baseline through four questions concerning participants' expectations about weight‐loss, satisfaction with appearance, and expected feelings of well‐being and less worries 3 years after surgery. Response options ranged from 1 to 10, with higher mean scores indicating higher expectations. Two separate mean scores were calculated: 1. Expectations about weight‐loss and appearance (EXP‐body), and 2. Expected feelings about well‐being and less worries (EXP‐mental health).

*Resilience* was assessed at baseline using the Resilience Scale for Adults, consisting of 33 items with 5 response options on each [63]. The scale covered various domains including personal strength (10 items reflecting perception of self and perception of the future), social competence (6 items), structural style (4 items), family cohesion (6 items), and social resources (7 items). A total mean score was calculated, with higher scores indicating greater levels of resilience (Cronbach's alpha 0.84).

*BMI* (kg/m^2^) pre‐MBS was measured using a platform scale (Seca 635, III; 0–300 kg).

*Employment status* was coded as “not employed” and “employed”. *Education status* was categorized as “lower secondary,” “higher secondary,” and “University/equivalent; 12 years or more”.

### 2.3 Ethics

The study was approved by the Regional Research Ethics Committee (2009/1248a) and the local data protection officer at the Oslo University Hospital (19‐2010 OUS‐A). All participants received written and oral information about the study by the surgeons before they submitted a signed consent form to the Department of Psychology at the University of Oslo.

### 2.4 Statistical Analysis

Mean group differences were examined using independent‐sample t‐tests. Changes in depressive symptoms from pre‐surgery to 5 years post‐surgery were analyzed using repeated measures analysis of covariance. Regression analyses, including simple and multiple logistic analysis, were conducted to identify variables predicting probable depression 5 years post‐surgery, separately for men and women. Simple logistic regression analysis encompassed all baseline variables. Baseline levels of depressive symptoms were included as a covariate in the multiple logistic regression model. Only study variables that significantly predicted the outcome variable in the simple logistic regression analysis were retained in the final multiple logistic regression models. Because of the small number of men in the sample, analyses were conducted using 2000 bootstrap samples with a 95% confidence interval.

## 3 Results

The sample consisted of 77.6% women (*n* = 163) and 22.4% men (*n* = 47). The mean BMI at baseline was 44.3 kg/m^2^ (SD = 5.74) and did not differ by gender (*p* = 0.624). The mean age for women was lower, and there were no significant gender differences in the level of education or employment status before surgery (Table 1).

The differences in mean scores for the study variables across genders are summarized in Table 1. Prior to surgery, there were no gender differences in depressive symptoms (*p* = 0.280). Women, however, reported higher body dissatisfaction (*p* = 0.003) and were more preoccupied with their appearance (*p* = < 0.001). Additionally, there were no significant gender differences in resilience (*p* = 0.439), expectations of weight/appearance change (*p* = 0.618), or expectations of reduced worry and improved well‐being (*p* = 0.077). Five years post‐surgery, men reported higher levels of depressive symptoms than women (*p* = 0.031).

**TABLE 1:** Gender differences in study variables before and 5 years after bariatric surgery.

	Women		Men			
	N	Mean (SD)	N	Mean (SD)	*t*	*p*
Pre‐surgery						
Age	163	43.4 (9.46)	47	47.6 (8.19)	−2.93	0.007
Education	158	0.33 (0.47)	46	0.26 (0.44)	0.91	0.376
Employed	157	0.73 (0.44)	45	0.78 (0.42)	−0.63	0.516
BMI	163	44.4 (5.46)	47	44.9 (5.96)	−0.50	0.624
Depressive symptoms	160	5.28 (3.62)	47	5.96 (3.83)	−1.08	0.280
BASS	163	2.38 (0.52)	47	2.71 (0.62)	−3.30	0.003
AO	163	3.62 (0.69)	47	2.85 (0.74)	6.36	< 0.001
EXP‐body	162	8.24 (1.62)	47	8.36 (1.42)	−0.49	0.618
EXP‐mental health	161	7.78 (1.86)	47	8.28 (1.64)	−1.77	0.077
Resilience	163	3.69 (0.63)	47	3.61 (0.63)	0.76	0.439
Five year post surgery						
Depressive symptoms	161	3.61 (3.35)	46	5.22 (4.37)	−2.31	0.031

*Note:* Group differences tested by independent‐samples‐*t*‐test.

Abbreviations: AO = appearance orientation; BASS = body area satisfaction; Education = level of education; Employment = employed or not; EXP‐body = expectancies of change in weight and appearance; EXP‐mental health = expectancies of change in worrying and feeling better; SD = Standard deviation.

Further exploration of depressive symptoms at the 5‐year mark included analyzing differences between participants classified as having probable depression versus no depression, as well as tracking changes in depressive symptoms across all time points (baseline, 1 year, and 5 years post‐surgery). Pre‐surgery, there were no significant gender differences related to probable depression classification (Table 2). However, at 1 year (*p* = 0.033), and 5 years (*p* = 0.021) post‐surgery, a higher proportion of men than women were classified as probably depressed.

In the overall sample, depressive symptoms decreased significantly from baseline to both 1 year and 5 years post‐surgery (Table 3). However, there was a significant increase in depressive symptoms from 1 year to 5 years post‐surgery. Both men and women showed a significant reduction in mean depressive symptom scores from pre‐surgery to 1 year post‐surgery. For women, depressive symptoms increased between one and 5 years post‐surgery, but remained lower at 5 years compared to baseline. For men, there was no significant change in depressive symptoms from baseline to 5 years post‐surgery.

For women (Table 4a), the simple logistic regressions showed that more depressive symptoms, greater body dissatisfaction, lower resilience, and higher expectations of change in weight and appearance at baseline, significantly predicted “probable depression” 5 years post‐surgery. In the multiple logistic regression analysis, only baseline depressive symptoms and resilience remained as unique predictors. For men (Table 4b), more depressive symptoms, and lower levels of resilience at baseline were significant predictors for probable depression at 5 years in both the simple and the multiple logistic regression models.

**TABLE 2:** Comparison of possible/probable versus no depression by gender.

Time point		Total sample % (*n*)	Women % (*n*)	Men % (*n*)	*X* ^2^	*p*
Pre‐surgery, baseline	No depression	71.1 (135)	72.8 (107)	65.1 (28)	0.95	0.344
	Probable depression	28.9 (55)	27.2 (40)	34.9 (15)		
1‐year post‐surgery	No depression	87.4 (166)	90.5 (133)	78.6 (33)	5.68	0.033
	Probable depression	12.6 (24)	9.5 (14)	23.3 (10)		
Five‐years post‐surgery	No depression	78.4 (149)	82.3 (121)	65.1 (28)	5.81	0.021
	Probable depression	21.6 (41)	17.7 (26)	34.9 (15)		

*Note:* Chi‐square test.

**TABLE 3:** Within group changes in depressive symptoms over time.

	Total sample	Women	Men
Depressive symptoms	(*n* = 190)	(*n* = 147)	(*n* = 43)
*Sig. Wilks Lamba*	< 0.001	< 0.001	0.015
Eta square	0.274	0.303	0.186
Baseline‐1 year	5.48–3.08^a^	5.28–2.74^a^	6.16–4.23^a^
1 year–5 years	3.08–3.95^a^	2.74–3.59^a^	4.23–5.19
Baseline‐5 years	5.48–3.95^a^	5.28–3.59^a^	6.16–5.19

*Note:* Repeated measure analysis of covariance.

^a^ Significant change.

**TABLE 4a:** Women: Probable depression five years after surgery—The association with variation in variables pre‐surgery.

Simple regression				Multiple regression		
				No depression (0) probable depression (1)		
No depression (0) probable depression (1)				(*n* = 158)		
	*n*	*p*	Exp (B)	*p*	Exp (B)	95% C.I. for Exp (B)
Baseline						
Depressive symptoms	158	< 0.001	1.342	0.004	1.229	1.06, 1.46
Not employed (0) versus employed (1)	155	0.492	0.741			
Education	156	0.339	1.530			
BMI	161	0.602	1.020			
Body satisfaction	161	0.037	0.347	0.867	1.143	0.28, 4.61
Appearance orientation	161	0.223	0.658			
Exp. Weight/app	160	0.046	0.777	0.415	0.892	0.67, 1.25
Exp. Worry/good	159	0.340	0.897			
Resilience	161	< 0.001	0.193	0.013	0.357	0.12, 0.81

*Note:* Simple and multiple logistic regression analysis.

**TABLE 4b:** Men: Probable depression five years after surgery—The association with variation in variables pre‐surgery.

Simple regression				Multiple regression		
				No depression (0) probable depression (1)		
No depression (0) probable depression (1)				(*n* = 46)		
	*n*	*p*	Exp (B)	*p*	Exp (B)	95% C.I. for Exp (B)
Baseline						
Depressive symptoms	46	0.003	1.504	0.022	1.395	1.04, 2.60
Not employed (0) versus employed (1)	44	0.146	0.360			
Education	45	0.090	0.308			
BMI	46	0.174	0.941			
Body satisfaction	46	0.230	0.509			
Appearance orientation	46	0.053	0.372			
Exp. Weight/app	46	0.888	0.965			
Exp. Worry/good	46	0.424	0.865			
Resilience	46	0.002	0.126	0.029	0.232	0.02, 0.91

*Note:* Simple and multiple logistic regression analysis.

## 4 Discussion

Both men and women experienced a decrease in depressive symptoms from pre‐surgery to 1 year post‐surgery. However, at 5‐years, improvement in depressive symptoms remained only for women. Across the total study sample, a significant rebound in symptoms was observed between one and 5 years post‐surgery. Specifically, 31.9% of men had scores indicative of depression pre‐surgery, decreasing to 22.7% at 1 year but increasing again to 34.8% at 5 years post‐MBS. For women, the respective proportions were 26.9%, 9.2%, and 17.4%. The proportion of men with probable depression was higher at both one and 5 years post‐surgery, despite no significant gender differences at baseline.

For women, several variables were found to be bivariate predictors of probable depression 5 years post‐surgery. However, in the multiple logistic regression model, only baseline depressive symptoms and lower scores on resilience remained as unique predictors. For men, both the simple and multiple analysis identified baseline depressive symptoms and lower resilience as unique predictors of probable depression 5 years post‐surgery.

The absence of gender differences in baseline depressive symptoms contrasts with typical findings in previous cohorts of candidates for MBS and the general population [11, 12, 23, 24]. However, one European study similarly reported no significant gender differences in pre‐surgery depressive symptoms [72], while one Asian study found no gender differences at 3–6 months post‐surgery [73]. A recent prospective study also found that gender did not predict depressive symptoms post‐surgery [74]. The present study aligns with prior research demonstrating a decline in depressive symptoms one and 2 years post‐surgery across the total sample [5, 14], as well as a more pronounced decline in symptoms among women [26]. Specifically, the pattern of an initial decrease followed by a rebound in depressive symptoms was consistent with earlier research [16, 18, 19, 33, 72, 75].

Unexpectedly, men scored higher on depressive symptoms, and a higher proportion met the criteria for probable depression 5 years post‐surgery. However, women scored higher on key risk factors of assumed relevance for depression, such as body dissatisfaction and appearance orientation. This finding aligns with previous studies [12, 49, 50, 54] and theories related to gender differences in weight and body image. Sociocultural explanations and research suggest that women are often socialized to prioritize their appearance [76, 77], engage more frequently with media body ideals, and experience greater stigmatization when overweight [78, 79].

For women, higher baseline weight and appearance expectations were a bivariate predictor of probable depression post‐surgery. This finding aligns with cross‐sectional studies that have linked unrealistic expectations to higher levels of depressive symptoms [80]. This study extends this observation by suggesting that such expectations may also predict depressive symptoms for women after surgery. Moreover, among women only, preoperative body dissatisfaction was associated with an increased likelihood of probable depression post‐surgery. This supports studies identifying body dissatisfaction as a key factor in depressive symptoms, noting that MBS involves complex body image changes [45, 46, 81, 82]. The present study adds that pre‐surgery body dissatisfaction is associated with probable depression post‐surgery, and that this is evident specifically among women. Although body dissatisfaction and expectations regarding changes in weight and appearance did not remain as significant predictors in the multiple model, this may indicate that body satisfaction, expectations, and resilience are interconnected. Notably, resilience appears to be particularly important in predicting probable depression post‐surgery in women.

For men, baseline depressive symptoms and resilience were bivariate predictors of probable depression 5 years post‐surgery, with both variables remaining as unique predictors in the multiple model. Unlike women, neither body satisfaction nor expectations of changes in weight and appearance were associated with probable depression post‐surgery. This suggests that, for men, body image may not play an equally significant role in predicting probable depression post‐surgery. Possible explanations could be that men tend to focus more on functional bodily aspects rather than appearance [83, 84, 85], and that men and women may experience depression differently [85, 86]. The relevance of resilience to depression was not surprising, as resilience encompasses successful adaptation to a stressor (MBS) involving both recovery and sustainability achieved through a range of intra‐ and interpersonal resources [87, 88]. Individuals with lower resilience may be more vulnerable to stressors after MBS and have greater difficulty adapting to necessary changes [87]. The importance of interpersonal resources, such as family cohesion and social support, aligns with reports finding that some patients experience challenging changes in close relationships, and stigma associated with MBS [66]. Research has supported the positive role of family cohesion, associating it with improvements in weight loss and healthy behaviors after surgery [89]. The association between resilience and probable depression supports previous cross‐sectional studies among patients following MBS [66, 67, 68]. Findings from this study suggest that resilience pre‐surgical resilience may serve as a buffer that protects against poorer outcomes after MBS, indicating that screening for resilience prior to surgery could enhance support provision.

Other factors, not explored in this study, may have influenced the likelihood of depression post‐surgery. The men in this sample were older than the women, indicating a longer wait before enrolling. Additionally, some studies have suggested that men typically present for surgery with more comorbidities compared to women [90]. Furthermore, fewer men than women, undergo body contouring surgery (skin removal) after MBS [91, 92], which has been associated with positive outcomes, including improved mental health [10, 93, 94].

### 4.1 Strength and Limitations

The majority of participants were women (77.6%), which aligns with the global prevalence of gender differences in undergoing MBS [95]. Because of the low number of men, bootstrapping techniques were employed to reduce the likelihood of Type‐I errors. Attrition analysis indicated no differences in drop‐out rates across genders; however, since we lack baseline characteristics on patients that were eligible for study inclusion but were not included, a selection bias cannot be ruled out.

The prospective and longitudinal study design allowed us to examine the relationship between pre‐surgery factors and post‐surgery factors within the same population. The single‐center design ensured consistency in surgical procedures and management practice, which enhanced the reliability of the findings, although it restricted generalizability.

Validated questionnaires were used for the evaluation of psychological symptoms and characteristics. Importantly, drop‐out rates regarding depressive symptoms from baseline to 5 years post‐surgery were not selective, indicating that participants who discontinued did not have significantly different baseline levels of depressive symptoms compared with those who remained in the study. HADS is not intended for diagnostic purposes; therefore, the results are not directly comparable with studies measuring clinical depression employing diagnostic interviews. With a suggested cut‐off point of ≥ 11 for identifying the likelihood of major depressive disorder (MDD), HADS has been shown to have higher specificity compared to sensitivity [96]. However, a meta‐analysis has suggested that heterogeneity observed in depressive symptoms post‐MBS cannot be explained by different depression instruments used across studies [17].

## 5 Conclusions

This study identified no significant gender differences in depressive symptoms prior to MBS and suggested that men may be more vulnerable to depressive symptoms following MBS. Furthermore, the results indicated that post‐MBS depressive symptoms may be associated with different factors dependent on gender. For women, body image and resilience were significant predictors, whereas for men, only resilience emerged as a predictor of probable depression after surgery.

## Funding

This work was funded by Department of Psychology, University of Oslo, Oslo, Norway.

## Conflicts of Interest

The authors declare no conflicts of interest.

## Data Availability

The data that support the findings of this study are available on request from the corresponding author. The data are not publicly available due to privacy or ethical restrictions.
